# The Diagnosis of the Internal Secretory Diseases

**Published:** 1916-09

**Authors:** Henry R. Harrower

**Affiliations:** M. D., F. R. S. M. (Lond.), Los Angeles, California


### TEXAS MEDICAL JOURNAL

**Dr. F. E. Daniel, Founder. Established July, 1885**

MRS. F. E. DANIEL, - Publisher and Managing Editor

Published Monthly.—Subscription, $1.00 a Year.

VOL. XXXII AUSTIN, SEPTEMBER, 1916 No. 3

**The publisher is not responsible for views of contributors**

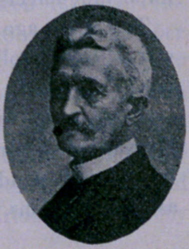

*"All of good the past hath had,*
*Remains to make our own time glad."*—WHITTIER.

### Original Articles.

### The Diagnosis of the Internal Secretory Diseases.

BY HENRY R. HARROWER, M. D., F. R. S. M. (LOND.), LOS ANGELES, CALIFORNIA.

### V.

### THE ADRENAL GLANDS IN HEALTH AND DISEASE.

Perhaps more profitable research has centered around the adrenal glands during the past ten years than around any of the other glands of internal secretion. At least many epoch-making discoveries of their important rôle have been made quite recently.

Unfortunately the clinical application of this new knowledge has not been very extensive as yet; and many times the physician's sole appreciation of adrenal disease consists of a hazy recollection that Addison's disease is said to be a tuberculous involvement of the adrenal glands—and that it is insurable.

Some things about the adrenals are very well known. We are in the habit of using adrenalin almost every day and know that it exerts a very decided influence upon the circulatory system, both in physiology and in therapy. We also know that the adrenin continuously produced by the adrenal medulla is the principal regulator of vascular tone and that it performs a number of other useful services for the body. But, somehow oı another, it is the exception to find a proper clinical appreciation of the importance of the work of the adrenals and how easily their functions may be influenced slightly or seriously with corresponding minor or important effects on the body as a whole.

Ten years ago T. R. Elliott, of London, showed us that adrenin virtually controlled the autonomic and sympathetic nervous systems. Sergent, of Paris, proved this relationship in numerous experiences in his clinical work. Still more recently Cannon, of Harvard, has given us an entirely new conception of the extreme importance of adrenin to the human economy especially in so far as its variations are related to the emotions.

SOME ADRENAL PHYSIOLOGY.

A brief exposition of the physiology of the adrenal glands will prepare us for a better understanding of their secretory disorders. The chromaffin hormone, otherwise known as adrenin, arising from the medullary portion of the adrenals, as well as in other chromophil cell collections in different parts of the body, exerts a very remarkable and extended influence upon numerous structures which are controlled by the sympathetic. Adrenin raises the blood pressure and has much to do with its maintenance at the average level; it dilates the pupils and excites the flow of tears and saliva; it contracts the minute muscles of the hairs (*erectores pilorum*); it undoubtedly is concerned with the function of the sweat glands, and, in fact, the blood supply of the skin, and, in addition to all this, it seems to have a certain influence upon the gastric, uterine and intra-abdominal muscles in general.

Adrenin is probably the principal factor in the maintenance of the normal tone of the body, and disturbances in its production disorganize the so-called "sympathetico-tonus," causing it to be deficient or abnormally increased as the case may be. The condition known as "adrenin sensibility" is now being used as the basis for several tests for sympathetic functioning which will be referred to later.

The adrenals are particularly susceptible to what have been termed the "emergency conditions." Cannon's well checked experiments have definitely proved that the emotions, including pain, rage, fear and hunger (perhaps it will be shown later that even worry has a similar effect) influence the secretory powers of the adrenals, with an immediate response due to the hyperadrenia this produced. This condition passes rapidly and as soon as the glands have been sufficiently overworked and the stimulation continues with no opportunity for recuperation, a serious condition of hypoadrenia supervenes.

While very little therapeutic advantage has been taken of the results of work, we can now see rational explanations for a number of phenomena which quickly can be called to mind. Practically the whole of the results of Crile's investigation of "the kinetic system" and his now fairly well known method of "anoci-association,"* are really dependent upon the prevention by suitable measures of any undue stimulation of the adrenal glands, and hence the serious consequences of acute hypoadrenia are thereby forestalled.

Before considering the symptomatology of the functional adrenal secretory dyscrasias, it should be recalled that not only are the emotional factors already referred to capable of causing this adrenal syndrome, but that certain of the hormones produced in other organs, when present in the blood stream in unusual amounts (see further references to this in the chapter on the ovaries) may have a similar stimulating effect. We must also remember that toxemia of intestinal or bacterial origin exerts a like influence and that it has been shown that conditions associated with extremely high blood pressure cause adrenal disorder, probably by producing intra-adrenal hemorrhages. One of the best established "symptoms" of senility is of adrenal origin.

With these facts in mind we can understand that severe emotional conditions, sudden or prolonged; acute infectious diseases with the invariable accompanying toxemia; and chronic infections, as tuberculosis or intestinal stasis (which is, after all, practically a chronic infection with mechanical involvement added) would be likely to bring about certain changes in the activities of the organism as a result of the influences due to adrenal derangement.

### HYPERADRENIA.

Hyperadrenia is not nearly so common a symptom as hypoadrenia, although it needs must be just as frequent, for the adrenal depletion of which we shall shortly speak is really a terminal condition which results from the exhaustion following excessive stimulation. The reason that hyperadrenia is not more commonly detected is probably due to the fact that adrenin is oxidized in the

*The kinetic system, according to Crile, embraces the adrenals, thyroid, brain and muscles, which cooperate to "drive" the body. The adrenals are probably the most important of these kinetic organs and the method of pan-anesthesia named "anoci-association" consists in supplementing the usual anesthetic measures by removing such mental and nervous stimuli (by preventing fear and pain and by "blocking" certain nerves) as would tend to stimulate the adrenals and by their depletion bring on shock and collapse.

blood with great rapidity, and that if large quantities of it happened to be brought forth, they are destroyed very shortly after they are produced. Confirmation of this destructive influence is noted following the use of adrenalin for therapeutic purposes, as well as in many experiments on animals which uniformly show that once this hormone gets into the blood it is very quickly destroyed.

However, it will be proper to enumerate several clinical findings which are probably of adrenal origin, since the treatment is largely preventive rather than direct, for to realize that certain factors are unduly stimulating the adrenals is to realize that these factors must be abated.

An unusual tendency to goose pimples, without any ordinary reason therefor, may be directly due to this condition. Probably this accounts for the not uncommon association of this phenomenon with fright. Chills, which are merely severe vaso-motor disturbances with muscular spasm, are commonly produced artificially by injections of adrenalin (especially following its use in the control of asthma), and I am by no means sure that this chief manifestation of malaria is not due to a temporary and excessive stimulation of the adrenal glands by the sudden unloosing of the toxins of the plasmodia. Further, the severe reaction following this positive phase of malaria, with its prostration, asthenia and depression simulates the symptom complex of hypoadrenia, as we shall shortly see.

This is a phase of the study of the adrenal glands that is not yet worked out, and this suggestion, which I believe is original, has neither been denied nor affirmed. Of course if this hypothesis is tenable, it would apply equally to pneumonia, typhoid fever and other acute toxemias where severe adrenal depletion is almost invariably an ultimate outcome.

In studying the relation of the adrenal glands to the toxemia of tuberculosis, Pottenger remarks that the continued stimulation of the adrenals and the continued pouring into the blood stream minutely increased amounts of adrenin, has the effect of producing a prolongation of the condition which is originally brought about by sympathetic stimulation. It is suggested that this condition of hyperadrenia is responsible for the dry mouth frequently seen in tuberculosis, and that other symptoms of sympathetic origin such as the sudden and seriously impaired digestion, and, particularly, the rapid heart action, are really the result of excessive adrenal stimulation.

It is quite possible that certain cases of purely functional hypertension, with no renal, cerebral or vascular findings demonstrable, are really due to hyperadrenia, usually of toxic origin. At least the interesting though academic researches of Zimmern and Cottenot, of Paris, seem to confirm this. They were able to reduce very high tensions by properly dosed roentgenization of the areas over the adrenals—to my mind a very serious undertaking.

There is still another form of hyperadrenia which must be mentioned though it is very rare. I refer to the condition known as "hypernephroma," which is an excessive proliferation of the adrenals usually involving the corticular tissue more than the medulla. The chief manifestation of this is a remarkable increase in the development and growth in early life (this is much more common in young subjects) with premature sexual development. Bullock, Sequeira and others have demonstrated a relation between the presumed internal secretion of the adrenal cortex and the gonads. At all events in cases of this disease the findings are chiefly referable to the gonad functions—a child of eight or nine may be quite as large as an adult with marked overdevelopment, physical and functional, of the genitalia, and hypertrichosis. It is a difficult, practically hopeless surgical condition.

(To be continued in October issue.)

